# Kawasaki Disease: Unusual Presentation with Retropharyngeal Involvement

**DOI:** 10.1155/2023/4913700

**Published:** 2023-04-24

**Authors:** Chiara Zeroli, Armela Gorica, Giulia Claire D'Aleo Canova, Monica Caruso, Paolo Castelnuovo, Francesca De Bernardi

**Affiliations:** Ospedale di Circolo e Fondazione Macchi, University of Insubria, Varese, Italy

## Abstract

**Background:**

Kawasaki disease is an acute febrile generalized vasculitic syndrome of childhood of unknown ethology. The most severe complication may involve the hearth and include acute myocarditis with hearth failure, arrythmia, and coronary artery aneurism. The typical clinical symptoms are fever, conjunctivitis, rash, cervical lymphadenopathy, and mucocutaneous changes, and the diagnosis is made by the clinical criteria. Early use of aspirin and immunoglobuline improves symptoms and prevent heart complications. *Case Presentation*. A 4-year-old male presented to our attention for multiple unilateral laterocervical lymphadenopathies, odynophagia, and neck stiffness, initially treated with IV antibiotic therapy with partial resolution of symptoms. After four months he made a new ER access for cervicalgia, tonsils asymmetry, trismus, stiff neck, lameness, and phalanx hyperaemia and increase in the size of cervical lymph nodes. Radiology showed increase of lymphnodes dimension and retropharyngeal space asymmetry. The same day heart murmur appeared, so the patient underwent cardiological evaluation that documented dilation of the coronary arteries. This sign made it possible to place the diagnostic suspicion of Kawasaki disease and to start IV immunoglobulins and acetylsalicylic acid administration with prompt response.

**Conclusions:**

Kawasaki disease presents with a range of symptoms which, taken individually, are very common in childhood. One of these symptoms is represented by the swollen of neck lymph nodes. It is only clinical reasoning that leads to the correct diagnosis, and therefore, to the correct setting of the therapy, reducing the risk of complications.

## 1. Introduction

Kawasaki disease (KD) is a childhood's acute febrile generalized vasculitis syndrome, first reported in 1967 in Japan by its namesake, Dr. Tomisaku Kawasaki [1]. While its cause is currently unknown, multiple theories have been proposed, ranging from genetic and environmental factors to an infectious etiology.

KD, as a multisystem disorder, involves predominantly medium and small-sized vessels, causing coronary artery aneurysms (CAA) thrombosis, stenosis, and even sudden death, making it one of the leading causes of acquired heart disease in developed countries. Occurring in young children, mostly below 5 years of age, with 1.5-times higher risk in boys than girls [2], KD is diagnosed by clinical criteria, reported in Table 1 [3].

It must be kept in mind that KD goes into differential diagnosis with many other diseases such as: toxic shock syndrome, Henoch–Schönlein purpura, exanthematous diseases such as measles, b-hemolytic streptococcus infection, toxic shock syndrome, Stevens-Johnson syndrome, and systemic juvenile idiopathic arthritis [4].

Retropharyngeal oedema is a rare presentation of KD that can be misdiagnosed as retropharyngeal abscess, leading to a wrong therapeutic attitude. Lameness occurs in about 33% of all cases, especially in the lower limbs and involving small joints [5]. Moreover, in some cases KD can be diagnosed as “incomplete” while referring to patients without enough diagnostic criteria in association with the typical fever or as “atypical” if concerning patients presenting early symptoms other than typical manifestations of KD (e.g., renal involvement, slowly resolving pneumonia, acute pancreatitis, and facial paralysis), in association to the disease's characteristic fever [6].

The most frequent blood count alterations associated with KD are represented by thrombocytosis and increased inflammatory indexes such as white blood cells and CRP.

Timely institution of treatment with intravenous immunoglobulin (IVIG) and high dose aspirin as early as possible after diagnosis causes rapid improvement of the disease and reduces the risk of CAA, while 10–20% of patients not treated in time develop these complications [7].

## 2. Case Report

A 4-year-old male presented to our attention referring left submandibular swelling accompanied by odynophagia and left flexion of the head, in absence of rhinitis or nasal obstruction. The patient was taken to the emergency room where blood chemistry tests were performed showing an increased level of WBC, CRP, and fibrinogen and normal levels of ALT, AST, and LDH. The SARS-COV-2 nasal swab test resulted negative. The neck ultrasound revealed multiple increased size lymph nodes in left II, III, and IV areas, with well recognizable hilum and no colliquation areas. The clinical examination highlighted a laterocervical mesoadenia and inspection of the oral cavity showed tonsils asymmetry (left > right), lateral oropharynx wall swelling and nasopharynx purulent discharge.

CT scan was performed in the clinical suspicion of parapharyngeal abscess, showing nasopharyngeal airspace asymmetry and multiple oval lymphadenopathies along the laterocervical stations bilaterally. Having evaluated both the clinical and radiological evidence, the multidisciplinary team decided to avoid surgery and proceed with IV antibiotic therapy.

**Figure 1:**
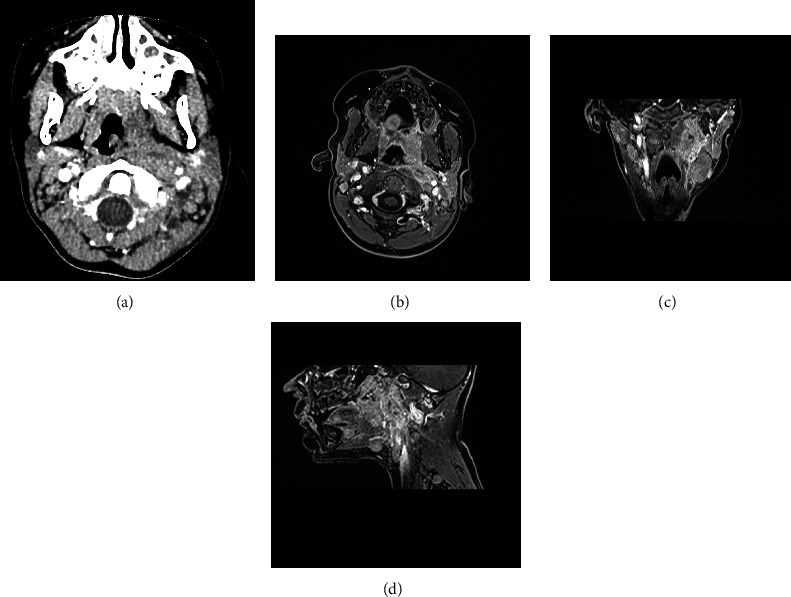
(a) Head and neck CT scan showing retropharyngeal soft tissue swelling and central low-density area, without rim enhancement. (b–d) Head and neck MRI (contrast enhanced T1) showing tissue enhancement with a central hypodense area of left deep parapharyngeal space near carotid space.

In view of an echographic increase in the lymphadenopathies size after medical therapy, a neck MRI was performed under sedation, confirming the persistence of obliteration on the left deep parapharyngeal and carotid spaces with left retropharyngeal lymphadenopathies (Figure 1).

The hospital's diagnostic protocol for pediatric lymphadenopathies was therefore applied, performing multiple tests to identify possible pathogens (blood culture, nasal secretion culture, intradermal Mantoux reaction, and serologies for EBV), which gave negative results.

Serology for Bartonella henselae resulted weakly positive (1 : 256) and the patient therefore perform an abdominal US that was within limits. Pharyngeal swab resulted positive for Group A beta-hemolytic *Streptococcus pyogenes* (GABHS).

During the patient's hospital stay, gradual reduction of the left cervical lymphadenopathies was documented, with symptoms abate and improvement of inflammatory indexes, except for persistence of a mild platelets level increase and the patient was then discharged at home with clarithromycin therapy.

After 4 months from the hospital discharge, the patient experienced a laterocervical swelling relapse, with left cervicalgia, stiff neck, trismus, and fever. At the same time, pain and swelling of the hands appeared, with polymorphous rush at the level of phalanx, foot-plant and lumbar region, and unilateral lameness in the absence of osteoarticular swellings. These symptoms then regressed spontaneously after some days. The SARS-COV-2 nasal swab test resulted negative, blood chemistry tests showed an increase in WBC, CRP, platelets, and fibrinogen, while blood cultures and throat swab cultures gave negative results. Concomitantly, a new antibiotic IV therapy was started and continued for 15 days. PCR and serology for Bartonella, Brucella, Borrelia, Toxoplasma, CMV, and Francisella were performed, all resulting negative. The neck US and MRI showed numerical and dimensional increase of the previously known laterocervical lymph nodes, with evidence of colliquation areas and retropharyngeal space asymmetry. No septic or meningeal signs were found at the clinical examination and the MRI did not highlight meningeal pathology, so it was not deemed necessary to assay cerebrospinal fluid.

Fibrolaryngoscopy revealed adenoid hypertrophy and a minimal asymmetry of the pharyngeal region without macroscopic swelling, with maintained airways patency.

**Figure 2:**
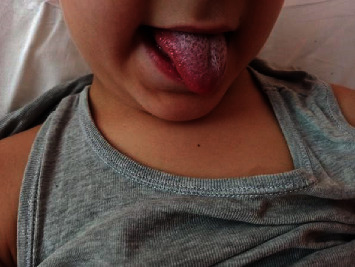
Strawberry tongue.

Contextually, the patient experienced strawberry tongue (Figure 2), conjunctival hyperemia and heart murmur. For this reason, cardiological evaluation and echocardiogram were performed, documenting coronary arteries dilation, wider at the level of the common trunk and the proximal right coronary artery (Z score 4.7 and 3.7, respectively).

Suspecting a potential KD diagnosis, the patient started the administration of intravenous immunoglobulins and acetylsalicylic acid at an anti-inflammatory dose.

**Figure 3:**
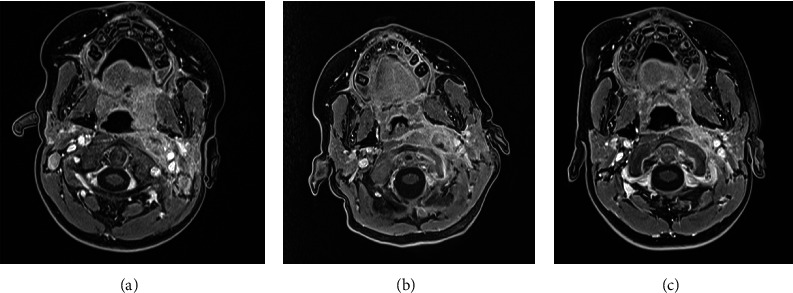
(a) Retropharyngeal space colliquation area on first admission. (b) Retropharyngeal space colliquation area on second admission. (c) Retropharyngeal space after treatment.

This therapy led to a prompt clinical and radiological response as follows: gradual improvement of the symptoms and the thermal curve, reduction in the caliber of the coronary arteries (Z score of 3.1 for the common trunk, 2.3 for the right coronary artery), reduction in the lymphadenopathies dimension at MRI (Figure 3), improvement of platelets level, and reduction of the CRP.

The patient continued IV antibiotic therapy for a total of 14 days and was then discharged in good clinical conditions on the 20^th^ day after the admission. Three months later the cardiologic control showed no cardiological sequelae and cardioaspirin therapy was stopped.

## 3. Discussion

Most childhood lymphadenopaties, around 80%, appear to be reactive, often having an infectious etiology. Other less common causes are: 14.3% miscellaneous (sarcoidosis, Gaucher disease, Niemann-Pick disease, Fabry disease hyperthyroidism, severe hypertriglyceridemia, and Kawasaki disease); 3.3% nonspecific reactive hyperplasia; 0.5% connectivity; and 2.3% neoplasm [4, 7].

In this case report, the pediatric-ENT protocol for management of cervical lymphadenopathies was applied, establishing the steps to follow to reach the diagnosis and start the adequate therapy.

In view of the pandemic period, the child was tested for SARS-COV-2 and resulted negative. This finding turns out to be relevant in view of the final diagnosis of KD for which the causes are not yet known.

The patient then underwent further investigations due to a nonresponse to antibiotic therapy: complete blood count, CRP, LDH, ALT/AST, EBV serology, neck ultrasound, CT scan, and MRI. Mantoux intradermal test resulted negative, with no change in injection site.

Due to the nonspecific results of these tests, second level diagnostic investigations were also performed, including serological investigations for Toxoplasma Gondii, Francisella Tularensis, Borrelia Burgdorferi, Brucella, and CMV. The patient tested positive at low titer for Bartonella H. Infection and had a positive pharyngeal swab for GABHS that warranted the clinical presentation on initial admission.

In addition to conjunctivitis, strawberry tongue, cutaneous rash, fever, and mesoadenia the patient manifested a cardiological symptomatology that allowed the diagnosis of Kawasaki disease. The blood count showed thrombocytosis, which frequently appears 15 days after the beginning of symptoms. Moreover, the patient manifested lameness, a symptom that, according to literature, may be present in 33% of KD cases [4].

In addition to classic KD symptoms, the patient also manifested stiff neck and retropharyngeal oedema, a quite rare symptom that could be misdiagnosed as retropharyngeal abscess leading to wrong therapeutic attitude [4].

Literature describes other cases of KD with retropharyngeal involvement, such as radiological findings at CT scan of increased density areas in the retropharyngeal space.

In view of the latency of 4 months between the two hospital accesses and considering that no other cases of KD with two episodes (with this temporal distance) are described in the literature, we do not exclude that the first episode may not be attributable to KD but instead to upper respiratory tract infections. However, the second episode presents the criteria for the diagnosis of KD and for these reasons we report this clinical case as relevant. These articles concluded that, in children presenting with fever and cervical lymphadenopathies that also display retropharyngeal low-density areas extending into deeper neck spaces, as well as changes in adjacent soft tissue, the hypothesis of KD should be considered [8].

## 4. Conclusion

The patient presented with a vague range of symptoms, such as persistent febrile cervical lymphadenopathies, stiff neck, lameness, and a radiological finding of retropharyngeal oedema.

To date, there are no specific tests to diagnose KD, hence, the importance to exclude other illnesses and make a differential diagnosis through first and second level tests, for which a clear protocol is necessary, in addition to a multidisciplinary evaluation of the clinical case. The interpretation of radiological analyses is also essential to define the correct therapeutic procedure and avoid unnecessary surgical procedures.

## 5. Disclosure

The research was performed as part of the employment of the authors at Ospedale di Circolo e Fondazione Macchi, Varese, Italy.

## Figures and Tables

**Table 1:** Criteria for the diagnosis of Kawasaki disease

Fever for more than five days (four days if treatment with IVIG eradicates fever) plus at least four of the following clinical signs not explained by another disease process
(1) Bilateral conjunctival injection
(2) Changes in the oropharyngeal mucous membranes (including >1 of the following symptoms: injected and/or fissured lips, strawberry tongue, injected pharynx)
(3) Changes in the peripheral extremities, including erythema of the hands and feet (acute phase) or periungual desquamation (convalescent phase)
(4) Polymorphous rash, primarily truncal; nonvesicular
(5) Cervical lymphadenopathy: anterior cervical lymph node at least >1.5 cm in diameter

Modified from Robert P. Sundel; Kawasaki disease, Rheum Dis Clin North Am 41 (2015) 63–73.

## Data Availability

The data used to support the findings of this study are included within the article..
